# Comparison of genovars and *Chlamydia trachomatis* infection loads in ocular samples from children in two distinct cohorts in Sudan and Morocco

**DOI:** 10.1371/journal.pntd.0009655

**Published:** 2021-08-09

**Authors:** Ehsan Ghasemian, Aleksandra Inic-Kanada, Astrid Collingro, Lamiss Mejdoubi, Hadeel Alchalabi, Darja Keše, Balgesa Elkheir Elshafie, Jaouad Hammou, Talin Barisani-Asenbauer

**Affiliations:** 1 Institute of Specific Prophylaxis and Tropical Medicine, Center for Pathophysiology, Infectiology and Immunology, Medical University of Vienna, Vienna, Austria; 2 Centre for Microbiology and Environmental Systems Science, Division of Microbial Ecology, University of Vienna, Vienna, Austria; 3 Institute of Microbiology and Immunology, Faculty of Medicine, University of Ljubljana, Ljubljana, Slovenia; 4 Federal Ministry of Health, Khartoum, Sudan; 5 Medicine and Pharmacy Faculty, Mohammed V University, Rabat, Morocco; Imperial College London, Faculty of Medicine, School of Public Health, UNITED KINGDOM

## Abstract

Trachoma is a blinding disease caused by repeated conjunctival infection with different *Chlamydia trachomatis* (Ct) genovars. Ct B genovars have been associated with more severe trachoma symptoms. Here, we investigated associations between Ct genovars and bacterial loads in ocular samples from two distinct geographical locations in Africa, which are currently unclear. We tested ocular swabs from 77 Moroccan children (28 with trachomatous inflammation-follicular (TF) and 49 healthy controls), and 96 Sudanese children (54 with TF and 42 healthy controls) with a Ct-specific real-time polymerase chain reaction (PCR) assay. To estimate bacterial loads, Ct-positive samples were further processed by multiplex real-time qPCR to amplify the chromosomal outer membrane complex B and plasmid open reading frame 2 of Ct. Genotyping was performed by PCR-based amplification of the outer membrane protein A gene (~1120 base pairs) of Ct and Sanger sequencing. Ct-positivities among the Moroccan and Sudanese patient groups were 60·7% and 31·5%, respectively. Significantly more Sudanese patients than Moroccan patients were genovar A-positive. In contrast, B genovars were significantly more prevalent in Moroccan patients than in Sudanese patients. Significantly higher Ct loads were found in samples positive for B genovars (598596) than A genovar (51005). Geographical differences contributed to the distributions of different ocular Ct genovars. B genovars may induce a higher bacterial load than A genovars in trachoma patients. Our findings emphasize the importance of conducting broader studies to elucidate if the noted difference in multiplication abilities are genovar and/or endemicity level dependent.

## Author summary

We investigated the association between different Ct genovars, the approximate load of infection, and the distribution of *Chlamydia* genovars by comparing samples from one trachoma-endemic area (i.e., the city of El-Gadaref in Al Qadarif, Sudan) and one previously endemic area (i.e., the Zagora Province in Morocco), currently considered as non-endemic. This study is the first to reveal a significant difference between the genome copy numbers of Ct genovar A and B/Ba in children with TF. Evidence that Ct is still circulating in rural foci of countries like Morocco that are no longer considered endemic implies that the continuation of the trachoma surveillance must be warranted in future to avoid further spreading of Ct. The clinical significance of different infectious loads in the development of sequelae has to be determined as well as whether these differences are genovar specific or related to the given endemicity level.

## Introduction

Trachoma is an infectious ocular disease caused by repeated infection of the conjunctiva with *Chlamydia trachomatis* (Ct) [1]. Worldwide, Ct is responsible for the visual impairment of approximately 2·2 million people, of whom 1·2 million are irreversibly blind [2,3]. Ct is the most prominent pathogen in the *Chlamydiae* phylum [4,5]. Differences between 19 serological variants (serovars) of Ct have been identified using monoclonal antibodies that react to epitopes on the major outer membrane protein (MOMP) [6,7]. Serovars A–C mainly cause trachoma, serovars D–K are major causes of sexually transmitted infections, and serovars L1–L3 mainly cause lymphogranuloma venereum, an invasive infection of lymph nodes [1,8,9]. Sequence heterogenicity in the outer membrane protein A (*ompA*) gene, which encodes MOMP, corresponds to 19 Ct genovars that reflect previously established serologic variants [10,11].

In trachoma epidemiology research, Ct typing helps to understand the temporal and geographical distribution of strains in endemic and non-endemic regions and could have significant implications for understanding transmission and pathogenicity, as well as improving vaccine development [8,12]. The main antigenic target of Ct, which has been extensively studied and is considered the main candidate for vaccine development, is MOMP [13–15]. MOMP forms approximately 60% of the total protein content of the outer membrane of Ct elementary bodies and consists of four variable domains (VDs) [16,17]. Differences in the amino acid structures of these VDs, resulting from genetic polymorphisms, have been associated with disease severity in several studies [18,19].

Regarding Ct infection in the urogenital tract, it has been suggested that the serovar class is related to the bacterial load [20,21] and that the infection load may influence Ct transmission within a population [22]. Assessment of the Ct infection load is also important in ocular samples because it can reveal useful relationships between the bacterial load and the clinical phenotype, risk of transmission, and maintenance of infection in a population [22–25]. Quantitative test results might be especially useful for identifying communities requiring more intensive treatment than standard annual mass therapy [23,26]. Previous data showed that the chance of repeated positivity and failed antibiotic therapy increased in patients with higher rectal Ct loads [27]. Moreover, a study published by Michel et al. in 2011 revealed that the infection load varies in areas with different levels of endemicity [28].

**Fig 1:**
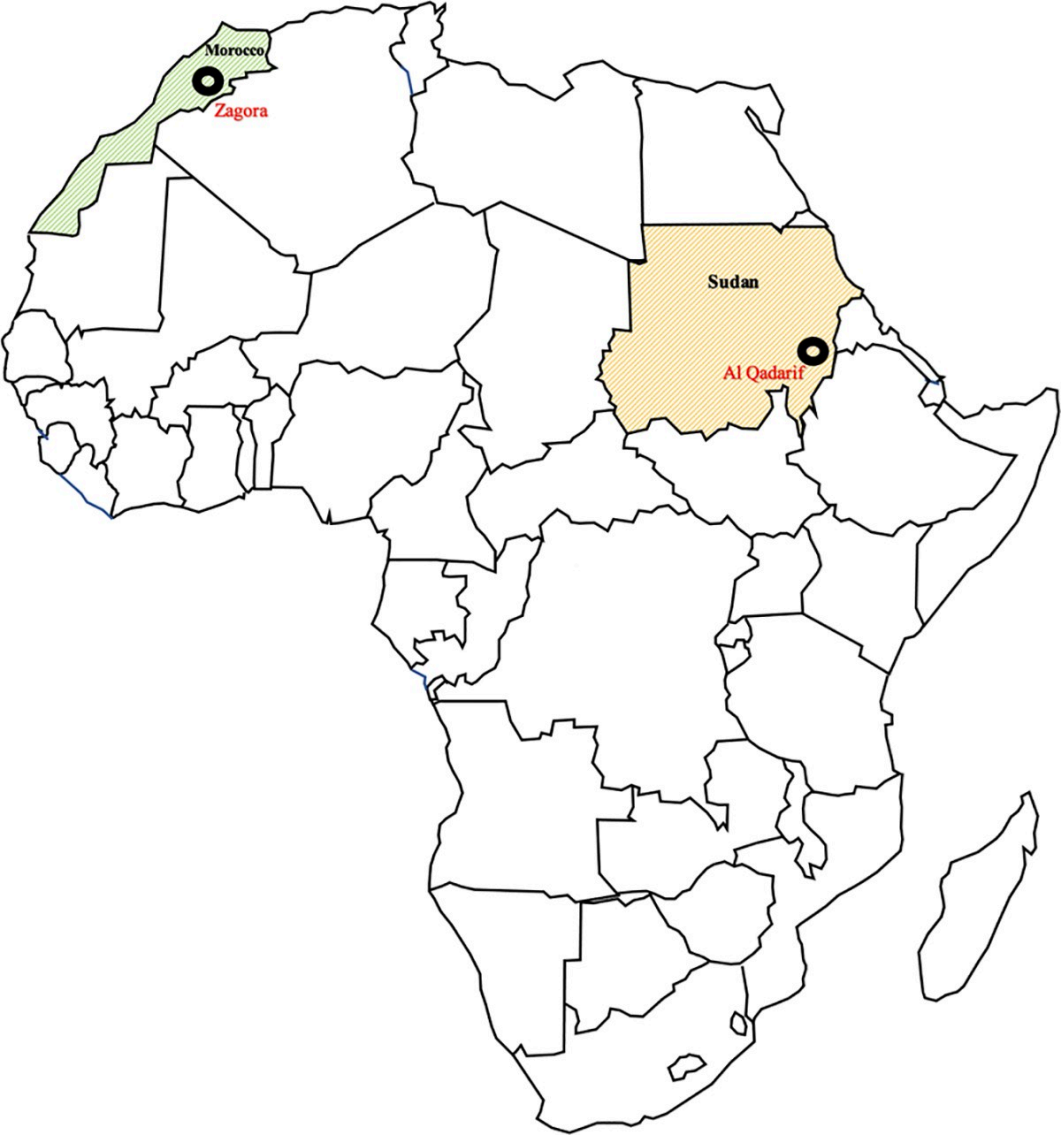
Location of the sampling sites. The map shows Zagora, a province in the Drâa-Tafilalet area of Morocco and the city of Al Qadarif, which is the capital of the state Al Qadarif in central Sudan. Direct link to the base layer map: https://www.statsilk.com/maps/download-free-shapefile-maps.

In this study, we aimed to explore the association between different Ct genovars, the approximate load of infection, and the distribution of *Chlamydia* genovars by comparing samples from one trachoma-endemic area (i.e., the city of El-Gadaref in Al Qadarif, Sudan) and one previously endemic area (i.e., the Zagora Province in Morocco) (Fig 1).

## Methods

### Ethics statement

This study was conducted in accordance with the Declaration of Helsinki. Written or verbal informed consent was obtained from a parent or guardian of all participants at the time of sample collection. When written consent was not possible, verbal consent for trachoma examination and sampling was documented by examiners on the data-collection forms. The study protocols, sampling methods, and consent procedures were approved by the National Ethics Authorities in Morocco (Moroccan Ministry of Health, Reference Number 1462 DELM/33) and Sudan (Sudanese National Research Ethics Review Committee, Reference Number 174-8-12). Personal identifiers were removed from the datasets before analysis. All samples were coded and anonymized. The Institutional Review Board of the Medical University of Vienna approved the data analyses presented in this study (Approval Number 2254/2017).

### Study population

In Morocco, the prevalence of trachomatous inflammation—follicular [TF] cases was successfully reduced to <5% in children from 1 to 9 years old in 2005. Since then, regular epidemiological surveillance has been conducted by the Ministry of Health. In this study, sampling was conducted after routine epidemiological surveillance for trachoma in children aged 1–15 years during the spring of 2013 by Moroccan government officials in previously endemic areas in the Zagora Province. In a previous surveillance study, the percentage of screened children with TF was 0·9–1·1% in this province [29]. A team of health care workers from the delegation of the Ministry of Health in the Zagora Province actively looked for symptomatic children by conducting sampling in several schools across the Zagora Province.

In 2012, health officials of the Sudanese Ministry of Health selected the city of El-Gadaref in Al Qadarif for sampling, where shortly before an epidemiological survey on the prevalence of trachoma was conducted [30]. In the state of Al Qadarif in Sudan, the prevalence of TF in children aged 1–9 years in rural regions was found to be between 5% and 19%. Screening of children in two madrassahs (Islamic religious schools) in rural regions of Al Qadarif was organized by the Sudanese National Program for Prevention of Blindness.

### Trachoma grading and conjunctival swabs

The recruitment of participants and sampling procedures were previously described by Ghasemian et al., 2018 [31]. Briefly, subjects were enrolled and screened for active trachoma in madrassahs by doctors and ophthalmic medical assistants trained in using the simplified grading system developed by the World Health Organization (WHO). Only eyes with ≥ 5 follicles with ≥ 0.5mm in the upper lid were graded as TF. None of the children was classified as TI.

Individuals with no clinical signs of conjunctival hyperemia, follicles, papillary hypertrophy, or conjunctival scarring were considered as control subjects. Conjunctival swabs were taken from the upper tarsal conjunctiva with polyester flocked swabs using the UTM-RT Collection Kit (Copan USA, Murrieta, CA, USA) using standard methods [32,33]. Swabs were stored in universal transport medium and frozen immediately in liquid nitrogen-cryogenic shipping containers. After the samples were received in Austria, they were stored at -80°C.

### Detection of Ct

Samples were screened for Ct using a multiplex real-time polymerase chain reaction (PCR) assay with two sets of primers and probes to detect plasmid open reading frame (pORF2) of Ct and the *Homo sapiens* RNase P/MRP 30-kDa subunit (*RPP30*) gene as an endogenous control (Table 1) [25,34].

**Table 1:** Primers and probes used for PCR reactions.

Target	Sense primer, antisense primer, and probe sequences (5′–3′)	Length (bp)	Reference
***omcB***	GAC ACC AAA GCG AAA GAC AAC AC ACT CAT GAA CCG GAG CAA CCT [FAM]-CCA CAG CAA AGA GAC TCC CGT AGA CCG-[BHQ1]	106	Pickett et al. [34]
**pORF2**	CAG CTT GTA GTC CTG CTT GAG AGA CAA GAG TAC ATC GGT CAA CGA AGA ^a^[FAM]-CCC CAC CAT TTT TCC GGA GCG A-[BHQ1] ^b^[HEX]-CCC CAC CAT TTT TCC GGA GCG A-[BHQ1]	109	Pickett et al. [34]
***H*. *sapiens RPP30***	AGA TTT GGA CCT GCG AGC G GAG CGG CTG TCT CCA CAA GT [HEX]-TTC TGA CCT GAA GGC TCT GCG CG-[BHQ2]	58	Butcher et al. [25]
***ompA***	ATG AAA AAA CTC TTG AAA TCG G ACT GTA ACT GCG TAT TTG TCT G	_~_1120	Jurstrand et al. [35]
***ompA*-inner**	TTG AGT TCT GCT TCC TCC T ACT GTA ACT GCG TAT TTG TCT G	_~_1100	Jurstrand et al. [35]

^a^*Ct* plasmid probe used for screening

^b^*Ct* plasmid probe for quantitative assays

Multiplex real-time PCR was performed in a final reaction volume of 20 μL, using 10 μL of iTaq Supermix (Bio-Rad, Reinach, Switzerland), primers and probes at final concentrations of 0·5 μM, and 5 μL of sample DNA. Thermocycling was performed with a PikoReal Real-Time PCR System (Thermo Fisher Scientific), using the following thermocycling conditions: 5 min at 95°C, followed by 45 cycles of 95°C for 15 s and 60°C for 40 s. All samples were tested in duplicate. Samples with a cycle threshold (C_T_) value of ≤37 were considered positive. DNA/RNA-free water was included as a negative control in each experiment.

### Estimation of chlamydial infection load

To estimate Ct infection loads, samples positive for Ct were further processed by performing another multiplex real-time PCR assay to amplify the chromosomal outer membrane complex B (*omcB*) gene and pORF2 of Ct (Table 1) [34]. Multiplex real-time PCR was performed in a final reaction volume of 20 μL, using 10 μL of iTaq Supermix (Bio-Rad, Reinach, Switzerland), each primer and probe at a final concentration of 0·3 μM, and 8 μL of sample DNA. The thermocycling conditions were 5 min at 95°C, followed by 45 cycles of 95°C for 15 s and 60°C for 40 s, and the reactions were carried out in a PikoReal Real-Time PCR System (Thermo Fisher Scientific). All samples were tested in duplicate. Two standard curves were drawn using 10-fold dilutions of the *omcB* gene and pORF2. The average Ct and plasmid loads for each sample were evaluated based on the C_T_ values for the *omcB* gene and pORF2, as described by Pickett et al., 2005 [34]. Because only one copy of the *omcB* gene is present on the Ct chromosome, the estimated copies of *omcB* correspond to the Ct infection-forming units (IFUs)/swab [34].

### Genotyping of Ct *ompA*

For Ct genotyping, a set of primers targeting the *ompA* gene (~1120 base pairs [bp]) was used for those samples previously tested positive for Ct pORF2 (Table 1) [35]. PCR-based genotyping was performed in a final reaction volume of 50 μL using 5 μL of AmpliTaq Gold buffer, 1 μL of dNTP mix, 5 μL of each primer, 4 μL of MgCl_2_, 0·3 μL of AmpliTaq Gold polymerase, 19·7 μL of ddH_2_O, and 10 μL of sample DNA. The thermocycling conditions used were 95°C for 10 min, followed by 40 cycles of 95°C for 30 s, 55·1°C for 30 s, and 72°C for 1·5 min, with a final step at 72°C for 7 min. All reactions were carried out in a Prime PCR System (Techne, UK).

After gel electrophoresis, PCR products were purified with the QIAquick PCR Purification Kit (Qiagen GmbH) according to the manufacturer’s protocol. Sanger sequencing of the purified PCR products was performed by Eurofins Genomics AT (Vienna, Austria) and Microsynth Austria GmbH (Vienna, Austria), using a pair of inner primers for the *ompA* gene (Table 1) [35].

### Data analysis

The Geneious R11.1.5 software package was used to trim, assemble, and align the obtained partial forward and reverse sequences of *ompA*. All consensus sequences were checked for the presence of chimeras with DECIPHER90 and compared with sequences available in the GenBank database of the National Center for Biotechnology Information (NCBI), using the BLAST-n server (https://www.ncbi.nlm.nih.gov/blast/). All coding sequences were translated into their corresponding amino acid sequences. Nucleotide and amino acid sequences were aligned to the reference sequences from the NCBI database, using Geneious R11.1.5 software to detect differences.

A p-value of < 0·05 was considered to reflect a statistically significant difference. A Fisher’s exact test was performed to study the association between the two classifications region and genovar, as well as sex and age of the participants. Wilcoxon rank-sum test was used to examine any association between Ct infection load and genovars or two classification regions, as well as Ct infection load and sex or age of the participants.

## Results

### Study population

A total of 173 swabs were taken from children aged 1 to 15 years (median age: 7 years). We collected 77 samples (28 patient samples and 49 control samples) from children in Morocco and 96 samples (54 patient samples and 42 control samples) from children in Sudan. The distribution of individuals between the case and control groups and by trachoma grades, sex, and age is shown in Table 2.

**Table 2:** Baseline and trachoma grades of study participants in Zagora Province (Morocco) and Al Qadarif (Sudan).

Country	Morocco				Sudan			
	Patients (N = 28)		Controls (N = 49)		Patients (N = 54)		Controls (N = 42)	
	N	%	N	%	N	%	N	%
**Sex** **Male** **Female**								
	11	39·3	26	53·1	31	57·4	22	52·4
	17	60·7	23	46·9	23	42·6	20	47·6
**Age** **≤5** **6-10** **11–15**								
	12	42·9	5	10·2	30	55·5	29	69
	9	32.1	13	26.5	24	44.5	13	31
	7	25	31	63.3	0	0	0	0
**Trachoma grade** **TF**								
	28	100	0	0	54	100	0	0

### Ct infection

Thirty-five (20·2%) out of 173 swabs taken from the subjects in this study tested positive for pORF2 by real-time PCR analysis. Ct positivity among the Moroccan and Sudanese children in the TF groups was 60·7% (17/28) and 31·5% (17/54), respectively (p = 0.011). One control subject out of 49 (2%) tested positive for Ct in Morocco.

### Distributions of Ct genovars

An *ompA* amplicon was successfully obtained from all samples positive for Ct, and each *ompA* amplicon was sequenced. Based on the BLAST results, 26 (74·3%) samples were classified as positive for genovar A and 9 (25·7%) samples were classified as positive for genovar B/Ba. There was no association between sex (p = 0.12) or age (p = 0.5) of the participants and Ct genovars.

Among the Sudanese cases, the prevalence of Ct genovar A (N = 16, 94.1%) was higher than that of genovar Ba (N = 1, 5.9%). However, among the Moroccan cases, positive samples for Ct were almost equally assigned to genovar A (N = 9, 52.9%) and B/Ba (N = 8, 47.1%). In general, the distribution of Ct genovar A and B/Ba was significantly different between Moroccan and Sudanese children (p = 0.018) (Table 3).

**Table 3:** Distributions of different *C*. *trachomatis* genovars among Sudanese and Moroccan children.

Characteristic	Overall	Morocco	Sudan	P-value
				0.018
**Genovar A**	26	10	16	
**Genovar B/Ba**	9	8	1	
**Tested negative**	138	59	79	

### Analysis of *ompA* sequences

Among 35 Ct-positive samples for which the *ompA* gene was sequenced, 32 (91·4%) samples exhibited nucleotide differences in the *ompA* gene sequence compared to the reference sequences. Positive samples for Ct genovar A were assigned to the A/HAR-13 and A/SA1/OT strains (Tables 4 and S1). Of the 24 samples positive for genovar A with nucleotide differences compared to their reference strains, 16 samples showed the highest similarity to strain A/HAR-13, and eight samples exhibited the highest similarity to strain A/SA1/OT (Tables 4 and S1). Changes in nucleotides 273 (A→C), 743 (C→T), 1098 (A→G), 1102 (G→A), and 1116 (T→C) were the most common polymorphisms among the genovar A-positive samples. We also found a nucleotide substitution at position 1098 (A→G) that was only present in the Sudanese samples positive for both A/HAR-13 and A/SA1/OT strains (Tables 4 and S1). The nucleotide change at position 743 (C→T) was identified as a specific polymorphism occurring only in the Moroccan samples positive for the A/HAR-13 strain (Tables 4 and S1).

**Table 4:** General profiles of *omcB* and plasmid copy numbers and BLAST-n analysis of the *ompA* gene for each Ct-positive sample.

Household ID	*omcB* count	pORF2 count	*ompA* genotype	Closest strain ^Ø^	Length (bp)	Phred score	Substitutions	Accession number
**Sudanese children, patients**								
**cTF-006**	343873	1876456	A	A/HAR-13	1097	50·5	A1098G, G1102A	
**cTF-008**	117672	768657	A	A/HAR-13	1085	50·8	A1098G, G1102A, T1116C	
**cTF-012**	5378	58474	A	A/HAR-13	1063	50·6	G736A	
**cTF-013**	14429	118127	A	A/HAR-13	987	48·6	G736A	
**cTF-017**	1109	7744	A	A/HAR-13	1064	50·2	-	DQ064279.1
**cTF-020**	126	1265	Ba	ATCC VR-347	1100	46·7	-	KP120856.1
**cTF-021**	195	1680	A	A/SA1/OT	1109	48·2	A273C, G736A, A1098G, T1116C	
**cTF-029**	2099	13301	A	A/HAR-13	1018	47·3	A1098G, G1102A, T1116C	
**cTF-031**	301477	2222022	A	A/HAR-13	1071	50·6	A1098G, G1102A	
**cTF-037**	2879	29940	A	A/SA1/OT	1111	48·9	A273C, A1098G, T1116C	
**cTF-038**	44152	255328	A	A/SA1/OT	1100	50·1	A273C, A1098G	
**cTF-044**	1481	6629	A	A/HAR-13	1017	43·6	A273C, G736A, C978T, A1098G, T1116C	
**cTF-048**	22421	243525	A	A/HAR-13	1016	43·8	A1098G, G1102A	
**cTF-057**	165672	1049077	A	A/HAR-13	1091	50·4	A1098G, G1102A, T1116C	
**cTF-059**	1635	9945	A	A/HAR-13	1099	50·8	A1098G, G1102A	
**cTF-068**	16243	131623	A	A/HAR-13	993	47·7	-	DQ064279.1
**cTF-088**	6382	74589	A	A/SA1/OT	1106	50·7	A273C, A1098G, T1116C	
**Moroccan children, patients**								
**cTF-103**	44152	262328	A	A/SA1/OT	1091	49·1	A273C, T1116C	
**cTF-117**	2227	16967	A	A/SA1/OT	1087	50·9	A273C, G742A	
**cTF-119**	20049	157984	B/Ba	(Ba/Apache-2) (B/Tunis-864)	1067	50·4	C429A, A511G/G760T	
**cTF-120**	97233	610794	B/Ba	(Ba/Apache-2) (B/Tunis-864)	1067	50·9	C429A, A511G/G760T	
**cTF-122**	20261	80348	B/Ba	(Ba/Apache-2) (B/Tunis-864)	1072	50·5	C429A, A511G/G760T	
**cTF-124**	1256799	10958227	B/Ba	(Ba/Apache-2) (B/Tunis-864)	1067	51	C429A, A511G/G760T	
**cTF-125**	1541130	12376436	B/Ba	(Ba/Apache-2) (B/Tunis-864)	1064	50·5	C429A, A511G/G760T	
**cTF-132**	31775	157984	A	A/HAR-13	1044	50·1	C743T, G1102A	
**cTF-135**	11090	69242	A	A/HAR-13	1093	51·3	C743T, G1102A, T1116C	
**cTF-136**	20719	152733	A	A/HAR-13	1091	51·2	C743T, G1102A	
**cTF-137**	1520985	8138389	B/Ba	(Ba/Apache-2) (B/Tunis-864)	1068	50·6	C429A, A511G/G760T	
**cTF-144**	3896	29142	A	A/SA1/OT	1091	50·1	A273C, G742A	
**cTF-148**	11090	64279	A	A/HAR-13	1047	48·2	C743T	
**cTF-149**	109457	512328	A	A/HAR-13	1094	50·5	C743T, G1102A	
**cTF-208**	360079	839278	B/Ba	(Ba/Apache-2) (B/Tunis-864)	1071	51	C429A, A511G/G760T	
**cTF-220**	41341	207047	A	A/HAR-13	1089	51·4	C743T, G1102A	
**cTF-222**	570692	4706407	B/Ba	(Ba/Apache-2) (B/Tunis-864)	1059	51	C429A, A511G/G760T	
**Moroccan children, control subjects**								
**cC-211**	3284	28173	A	A/HAR-13	1015	47·6	C743T	

Samples positive for Ct genovars B/Ba were assigned as the Ba/Apache-2, B/Tunis-864, and ATCC VR-347 strains (Table 4). With the samples positive for genovar B/Ba, it was not possible to differentiate between the Ba/Apache-2 and B/Tunis-864 strains, as they contained only one nucleotide polymorphism when compared to each of these reference strains (Table 4). Among the eight samples positive for genovar B/Ba with nucleotide differences compared to the reference strains, nucleotide substitutions at positions 429 (C→A), 511 (A→G, only in Ba/Apache-2), and 760 (A→T, only in B/Tunis-864) were most frequent (Tables 4 and S1).

### Genovars and infection loads

**Fig 2:**
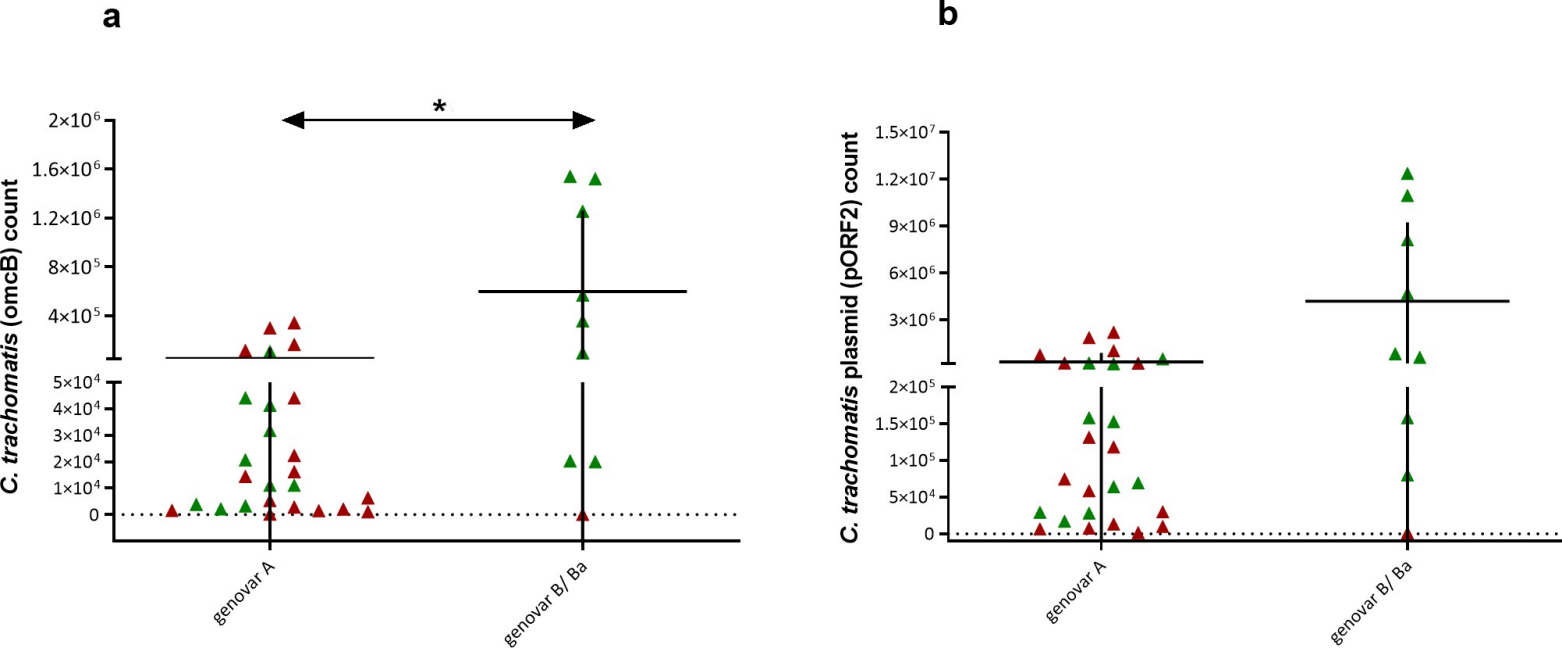
Bacterial loads and plasmid copy numbers for different Ct genovars. Scatter dot-plots show the mean Ct load (a) and plasmid copy number (pORF2) (b) in samples positive for the B/Ba genovars were significantly higher than those for genovar A. Samples from Moroccan children are marked in green and samples from Sudanese children are marked in red. The vertical lines indicate the error bars that express the standard deviations, and the horizontal lines indicate the means. Statistical significance is indicated as follows: *p = 0·017.

**Fig 3:**
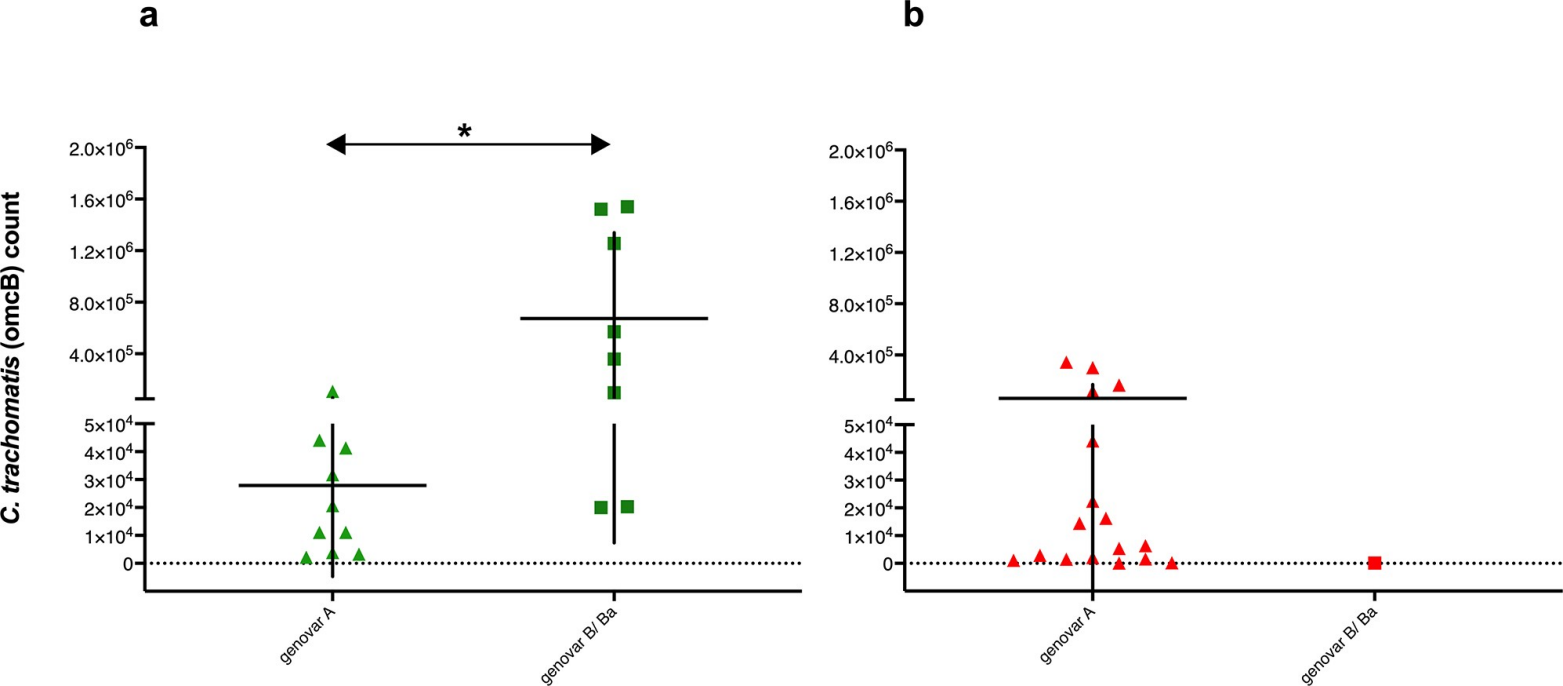
Bacterial loads for different Ct genovars among Moroccan and Sudanese children. Scatter dot-plots show the mean Ct load for genovar A and B/Ba among Moroccan (a) and Sudanese children (b). Moroccan samples are marked in green and samples from Sudan are marked in red. The vertical lines indicate the error bars that express the standard deviations, and the horizontal lines indicate the means. Statistical significance is indicated as follows: *p = 0·011.

Samples positive for the Ct were tested by performing multiplex real-time PCR detection of *omcB/*pORF2 to estimate the numbers of IFUs and Ct plasmids in each swab. We found significantly more (p = 0.017) of Ct *omcB* copies in samples positive for Ct genovar B/Ba (598596) than in those positive for genovar A (51005), as shown in Fig 2. We determined a significantly higher Ct infection load in samples from Moroccan children (314792 *omcB* copies) than in samples from Sudanese children (61601 *omcB* copies) (p = 0.032). When considering samples positive for genovar A in both communities, the infection loads of samples from Sudanese children (65443 *omcB* copies) was higher than those in samples from Moroccan children (27903 *omcB* copies) (p = 0.2). Among Moroccan children, the Ct load was significantly higher for samples positive for genovar B/Ba (673403 *omcB* copies) than in those positive for genovar A (27903 *omcB* copies) (P = 0.011) (Fig 3). In this study we did not find any association between sex of the participants and the infection load (p = 0.5) or the distribution of different Ct genovars (p = 0.12). Moreover, there was no association between age of the participants and the infection load (p = 0.15).

### Plasmid copy numbers per Ct

Dividing the plasmid copy number into *omcB* gene copy number for each sample, resulted in an average number of plasmids per chromosome of Ct. On average, we found 6·7 plasmid gene copies per Ct *omcB* gene. We did not find any significant difference (p = 0·93) between the number of plasmids per genome for Ct genovar A and genovar B/Ba.

## Discussion

In this study, we analyzed Ct bacterial loads and genovar diversities in ocular samples from children with or without signs of trachoma, who were living in one endemic region of Africa and in one previously endemic region.

Regarding geographical distributions, we found different positivity patterns for Ct and its genovars in symptomatic children from the Zagora Province of Morocco, compared to those from Al Qadarif, Sudan. Ct was found in 60·7% of symptomatic children from Morocco. In contrast, only 31·5% of symptomatic children in Sudan tested positive for Ct. The prevalence of Ct genovar A and B/Ba was significantly different between Sudanese and Moroccan cases. Although majority of Sudanese children cases (N = 16) were assigned to genovar A and only one sample to genovar Ba, the distribution of Ct genovar A (N = 9) and B/Ba (N = 8) was nearly equal between Moroccan children cases.

Differences in the prevalences of Ct genovars in trachoma samples, based on differences in the geographical zones, have been shown previously. For example, genovars C and Ba are more prevalent in Australia [36], and genovars A, B, and Ba are more prevalent in Africa [37–42]. Our results support the study by Takourt et al. [40], which demonstrated a high prevalence of the Ba (63%) and A (45%) genovars in Moroccan children with signs of trachoma. The distributions of Ct genovars in Sudanese children were comparable with those reported for the neighboring country, Ethiopia, showing a higher prevalence of genovar A than other trachoma genovars [42,43]. Our results are consistent with a recent whole-genome sequencing study of 12 Ct samples from trachoma patients in Al Qadarif, Sudan, which showed that all tested samples were positive for Ct genovar A [44].

In this study, we found significantly higher plasmid and genome copy numbers in samples positive for Ct genovar B/Ba compared to samples positive for genovar A. These results support prior studies that suggested an association between Ct strain diversity and the infection load [41,44]. Data from a study by Jolly et al. [45] revealed significantly lower progeny production with Ct genovar A when compared with genovar Ba in primary ocular and endometrial stromal fibroblast cells. To our knowledge, our current study is the first to reveal a significant difference between the genome copy numbers of Ct genovar A and B/Ba in children with TF. Results from previous studies by West et al. and Last et al. suggested that the infection load might be an essential factor in Ct transmission [22,46].

Based on the finding of approximately two-fold higher positivity of Ct infection in Moroccan patients than in Sudanese patients, we speculate that genovars among the Moroccan study population might have higher transmission potential compared with genovars in the Sudanese study population. The successful implementation of the surveillance strategies by the Moroccan government could efficiently prevent the widespread dissemination of these genovars. At the time of sampling in Morocco, we found that Ct was still circulating in an area that was no longer considered endemic for trachoma. Yet, it has to be noted that symptomatic cases are rare and were only detected by rigorous trachoma surveillance in the region. It is possible that asymptomatic Ct infections (in adults and children) were not reached by the elimination program and that a reservoir of infections exists in certain rural pockets [29,47]. Moreover, at the time this study was conducted, not all families in the study region had access to drinking water, and the resulting lack of adequate hygiene practices might have sporadically contributed to local foci formation. Therefore, we suggest that the association between sporadic cases of TF and occasional inadequate environmental hygiene should be further investigated. Our findings indicate that we should not underestimate the risk of trachoma reemergence in previous endemic counties. Noteworthy, by the end of 2005, Morocco achieved the epidemiological end-points defined by the WHO for eliminating trachoma as a public health problem. Moreover, Morocco has succeeded in controlling trachoma by implementing all four interventions of the SAFE strategy (surgery for trichiasis, antibiotics, facial cleanliness, and environmental improvement) [1,29].

The impact of the Ct infection load on disease severity in trachoma has been reported in several studies [22,48,49]. Data from two studies, one on patients with trachoma by Bobo et al. [50] and another on non-human primates by Kari et al. [51], suggested a link between different Ct genotypes and the disease severity and duration of infection. In a prior study by Chin et al. genovar B was reported to cause more severe symptoms of intense trachomatous inflammation than genovar A [42]. Results from Last et al. [22] and Andreasen et al. [41] further suggested a connection between different Ct genotypes and the infection load in patients with trachoma.

We found geographically distinct mutations in the *ompA* gene of Ct strains A/HAR-13 and A/SA1/OT in Sudanese children compared to those in Moroccan children. Our finding is consistent with previous studies on patients with trachoma from distinct geographical locations, showing unique diversities in the *ompA* gene sequence at each sampling site [41,43,52]. We detected four non-synonymous mutations among patients positive for genovar A, and two in both B (B/Ba) Ct genovars (S1 Table). This finding is consistent with other studies demonstrating evidence of the slow diversification of Ct [26,44,53,54].

Limitations to this study is that the sampling in Morocco and Sudan was done with the aim to collect different Chlamydia isolates in the context of our vaccine development, e.g., the sampling was not intended to be a large epidemiological study. Due to the small sample size and low number of observations with genovar B it is not possible to adjust for confounding variables like age, region or load on genovar. For validation of our findings studies with larger study populations are required.

Previous findings revealed an average of five plasmid copies per Ct chromosome. However, a wider range from one to 18 plasmid copies per chromosome has been reported [25,34]. These studies are consistent with our findings, which showed 6·7 plasmid copies per Ct chromosome. On average, we found a similar plasmid copy number per chromosome for genovars A and B/Ba, which support the results from Pickett et al., 2005, which indicated that no significant difference occurred among different strains of Ct [34].

In conclusion, geographical differences were found to contribute to the distribution of different trachoma Ct genovars. Genovars B/Ba may induce higher bacterial loads than genovar A in the eyes of subjects with trachoma. Further *in vitro* studies may help to elucidate the link between different Ct genovars and differences in their multiplication abilities. Regarding Ct circulation in the pockets, trachoma surveillance programs need to be continued even in countries considered non-endemic, especially in rural regions with poor hygiene practices.

## Supporting information

S1 Table List of nucleotide polymorphisms and amino acid diversity in the *ompA* gene of positive samples for *C*. *trachomatis* (Ct). Positions and abbreviations of the amino acids corresponding to the nucleotides before and after polymorphisms are mentioned in brackets.
(DOCX)
