# Supplementary material for: Comparison of genovars and Chlamydia trachomatis infection loads in ocular samples from children in two distinct cohorts in Sudan and Morocco
Source: PLoS Negl Trop Dis. 2021 Aug 9;15(8):e0009655. doi: 10.1371/journal.pntd.0009655 (PMC8376198; doi:10.1371/journal.pntd.0009655)
Supplement: S1 Table — Positions and abbreviations of the amino acids corresponding to the nucleotides before and after polymorphisms are mentioned in brackets. (DOCX) [file pntd.0009655.s001.docx]

**Supporting information**

| **Ct Strain** | **Household ID** | **Genovar A** | | | | | | **Genovar Ba** | | **Genovar B** | |
| --- | --- | --- | --- | --- | --- | --- | --- | --- | --- | --- | --- |
|  |  | **273 A**  **(91Arg)** | **736 G**  **(246Val)** | **743 C**  **(248Ala)** | **1098 A** | **1102 G**  **(368Val)** | **1116 T** | **429 C**  **(143Ser)** | **511 A**  **(171Ser)** | **429 C**  **(143Ser)** | **760 G**  **(254Ala)** |
| **Strain A/HAR-13** | **Sudan** | | | | | | | | | | |
|  | cTF-006 |  |  |  | G | A (Ile) |  |  |  |  |  |
|  | cTF-008 |  |  |  | G | A (Ile) | C |  |  |  |  |
|  | cTF-012 |  | A (Ile) |  |  |  |  |  |  |  |  |
|  | cTF-013 |  | A (Ile) |  |  |  |  |  |  |  |  |
|  | cTF-029 |  |  |  | G | A (Ile) | C |  |  |  |  |
|  | cTF-031 |  |  |  | G | A (Ile) |  |  |  |  |  |
|  | cTF-048 |  |  |  | G | A (Ile) |  |  |  |  |  |
|  | cTF-057 |  |  |  | G | A (Ile) | C |  |  |  |  |
|  | cTF-059 |  |  |  | G | A (Ile) |  |  |  |  |  |
|  | **Morocco** | | | | | | | | | | |
|  | cTF-132 |  |  | T (Val) |  | A (Ile) |  |  |  |  |  |
|  | cTF-135 |  |  | T (Val) |  | A (Ile) | C |  |  |  |  |
|  | cTF-136 |  |  | T (Val) |  | A (Ile) |  |  |  |  |  |
|  | cTF-148 |  |  | T (Val) |  |  |  |  |  |  |  |
|  | cTF-149 |  |  | T (Val) |  | A (Ile) |  |  |  |  |  |
|  | cTF-220 |  |  | T (Val) |  | A (Ile) |  |  |  |  |  |
|  | cC-211 |  |  | T (Val) |  |  |  |  |  |  |  |
| **Strain A/SA1/OT** | **Sudan** | | | | | | | | | | |
|  | cTF-021 | C (Ser) | A (Ile) |  | G |  | C |  |  |  |  |
|  | cTF-037 | C (Ser) |  |  | G |  | C |  |  |  |  |
|  | cTF-038 | C (Ser) |  |  | G |  |  |  |  |  |  |
|  | cTF-044 | C (Ser) | A (Ile) |  | G |  | C |  |  |  |  |
|  | cTF-088 | C (Ser) |  |  | G |  | C |  |  |  |  |
|  | **Morocco** | | | | | | | | | | |
|  | cTF-103 | C (Ser) |  |  |  |  | C |  |  |  |  |
|  | cTF-117 | C (Ser) |  |  |  |  |  |  |  |  |  |
|  | cTF-144 | C (Ser) |  |  |  |  |  |  |  |  |  |
| **Strain Ba/ Apache-2**  **or**  **B/Tunis-864** | **Morocco** | | | | | | | | | | |
|  | cTF-119 |  |  |  |  |  |  | A (Arg) | G (Gly) | A (Arg) | T (Ser) |
|  | cTF-120 |  |  |  |  |  |  | A (Arg) | G (Gly) | A (Arg) | T (Ser) |
|  | cTF-122 |  |  |  |  |  |  | A (Arg) | G (Gly) | A (Arg) | T (Ser) |
|  | cTF-124 |  |  |  |  |  |  | A (Arg) | G (Gly) | A (Arg) | T (Ser) |
|  | cTF-125 |  |  |  |  |  |  | A (Arg) | G (Gly) | A (Arg) | T (Ser) |
|  | cTF-137 |  |  |  |  |  |  | A (Arg) | G (Gly) | A (Arg) | T (Ser) |
|  | cTF-208 |  |  |  |  |  |  | A (Arg) | G (Gly) | A (Arg) | T (Ser) |
|  | cTF-222 |  |  |  |  |  |  | A (Arg) | G (Gly) | A (Arg) | T (Ser) |
